# Therapeutic options to treat mustard gas poisoning – Review

**DOI:** 10.22088/cjim.10.3.241

**Published:** 2019

**Authors:** Mehrdad Rafati-Rahimzadeh, Mehravar Rafati-Rahimzadeh, Sohrab Kazemi, Ali Akbar Moghadamnia

**Affiliations:** 1 Cancer Research Center, Health Research Institute, Babol University of Medical Sciences, Babol, Iran; 2 Department of Medical Physics, Kashan University of Medical Sciences, Kashan, Iran; 3 Cellular and Molecular Biology Research Center, Health Research Institute, Babol University of Medical Sciences, Babol, Iran

**Keywords:** Sulfur mustard, Mustard gas, Blistering (vesicant) agents, Bronchopneumonia, Chronic obstructive pulmonary disease.

## Abstract

Among the blistering (vesicant) chemical warfare agents (CWA), sulfur mustard is the most important since it is known as the “King of chemical warfare agents”. The use of sulfur mustard has caused serious damages in several organs, especially the eyes, skin, respiratory, central and peripheral nervous systems after short and long term exposure, incapacitating and even killing people and troops. In this review, chemical properties, mechanism of actions and their effects on each organ, clinical manifestations, diagnostic evaluation of the actions triage, and treatment of injuries have been described.

## Introduction

Over a few decades ago till now, due to abundant availability of various chemicals, the rate of intoxication has surprisingly increased (1, 2). People can overuse or misuse some chemicals, and get poisoned intentionally or accidentally (3, 4). An important point to pose is that chemical agents continue to be a concern used by terroristic organizations, and local- regional wars. These agents have seriously caused short and long-term damages, kill, or incapacitate ordinary and military persons in urban and war fields (5). The first reports of the use of chemical warfare agents have been found in ancient Greek and Roman writings. The modern uses of the agents have been reported during World War I (WWI). The Geneva Protocol in 1925 was the first major international effort to limit development, and subsequently use these agents during World War II(WW II), as well as their frequent use up to now (5, 6). Among the mass destruction weapons, chemical warfare is one of the most brutal created by mankind. In the last decades, modern chemical warfare agents have been repeatedly used in various classes with different chemical properties. They cause toxic and lethal effects and extensive human suffering (7). Blistering (vesicant) agents are important substances of CWAs. Sulfur mustard (mustard gas or “king of CWAs”), nitrogen mustards (HN1, HN2, and NH3) and lewisites (L1, L2, and L3) are the major categories of blister agents (8). The most commonly used chemical warfare agent is sulfur mustard (mustard gas). Other names are yperite (Y pres was the place of the first military use), LOST (the first family name of the German chemists’ **Lo**mmel and **S**teinkopf investigated the military use), and yellow cross (German shells were marked with a yellow cross due to skin damage (9). Sulfur mustard (SM) was first used on the battlefield near Ypres, Belgium on July 12, 1917 by German military forces. It was responsible for more than 80% of the recorded chemical warfare injuries. In December 1943, an allied ship carried a large amount of mustard gas and exploded in Bari harbor, Italy. It has been used sporadically since World War I (10), but the last military use was during Iran- Iraq war (1980-1988), specially occupied the village of Halbja in 1988 by Iraq chemical attack, wherein over 100000 of people and soldiers were injured and one- third of them have been suffering from its late effects so far(9, 11, 12).

■ **Physico-chemical properties**

Pure sulfur mustard [C_2_H_4_Cl_2_S] is a colorless and odorless liquid, but because the impure substance has smell similar to mustard or garlic. It is easily soluble in organic solvents and slightly in water (8). Some of physicochemical properties of sulfur mustard include; molecular weight: 159.08, density: 1.27 (specific gravity), solubility: very hydrophobic, freezing point: 14.45 °C, boiling point 215-217 °C (9). It has strongly alkylating, nucleophilic, lipophilic, cytotoxic, mutagenic and carcinogenic properties. It is known as the “king of the battle gases (5, 13).

■ **Mechanism of action**

**Fig 1:**
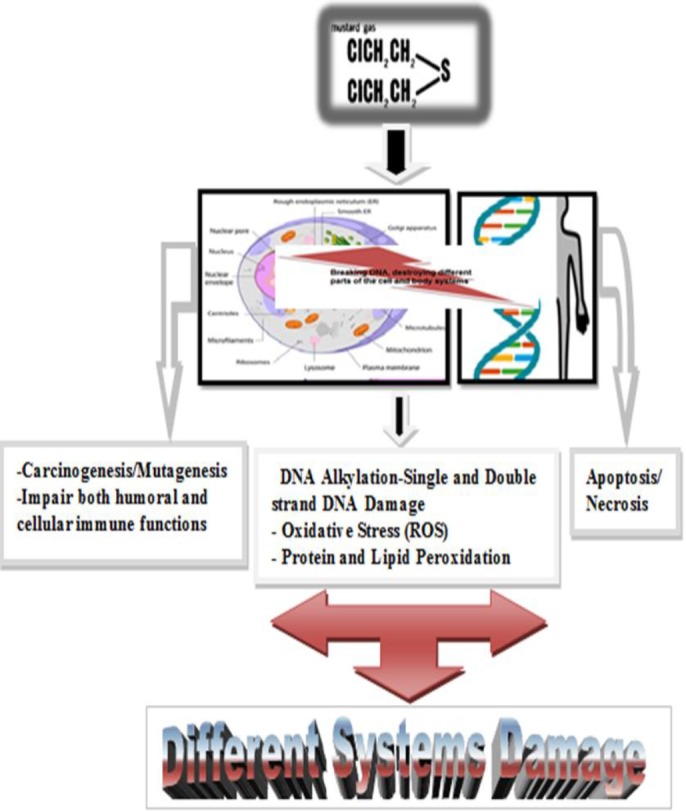
Scheme in relation to damaging various systems via mustard poisoning

A few theories explained the mechanism of action of sulfur mustard. The first was about the acid liberation theory; in which sulfur mustard becomes hydrolyzed within cells as hydrochloric acid. This theory was not soon accepted because vesicant action cannot act along with the amount released acid (9). Another theory was reactions of sulfur mustard with proteins and several enzymes that were inhibited, specially hexokinase. It is important that the level of alkylation needed for in vitro inhibition of these enzymes is not enough at vesicant doses in vivo. In addition, hexokinase may have inhibited alkylation after a few minutes. But, sulfur mustard appears to induce tissue damage with a noticeable delay in vitro and in vivo. Therefore, this theory may be rejected (9, 14, 15). Another theory suggests that sulfur mustard may deplete glutathione storage and lipid peroxidation. On the other hand, the sulfhydryl groups in proteins and other compounds containing glutathione can quickly deactivate lipid peroxidation processes. These compounds maintained the suitable oxidation- reduction reactions in cells. In fact, glutathione reduces reaction oxygen species in cell, also prevents peroxidative processes. When cells are exposed to sulfur mustard, depletion of glutathione and then lipid peroxidation occur. All the above mentioned theories are not in agreement with delay damage after sulfur mustard exposure. However, some of these theories justify and recognize sulfur mustard cytotoxicity (9, 11). The most important theory is alkylation of cell parts by sulfur mustard. That means alkylation reactions in affected cells, mainly are responsible of injuries of DNA, RNA, protein, lipid membranes. Mustard spontaneously undergoes intramolecular cyclization. This cyclization causes to eliminate chloride ion forming ethylene sulfonium ring. Reactive sulfonium ion alkylates sulfhydryl (-SH) and amino (-NH_2_) groups. That makes this point an indirect inhibition of glycolysis (16).The ethylene sulfonium in the middle of the process is converted to carbonium ion, and reacts immediately with DNA, RNA, protein and other molecules (9). At last, sulfur mustard reacts with DNA, which is the result of the N7 position of guanine [7- (2-hydroxyethylthioethyl)] guanine (7-HETE-G) (61%), N1 position of adenine, N3 position of adenine (16%) and O6 position of guanine (0.1%) (17, 18). The above description is a summary of the DNA alkalization. This process is achieved as single-and-double-strand DNA breaks. Then, cells try to repair the damaged DNA. This leads to activations of poly (ADP-ribose) polymerase (PARP) (19). Two classes are presented PARP-1 and PARP-2 in activated by DNA strand breaks. PARP-1 is a first line protein encountered in the cellular response to DNA strand breaks. The biological activity of PARP-1 causes to maintain survival and cell integrity undergoing genotoxic stress (20). But excessive PARP activity may cause to the cellular depletion of nicotinamide adenine dinucleotide (NAD^+^), also, simultaneous reduction of glycolysis (15). NAD^+ ^depletion and glycolysis inhibition lead to impairing energy production in the cell. As a result, ATP loss causes cell death (17) (Fig1).

Mustard gas changes in the different parts of the cell. DNA alkalization and DNA breakdown cause oxidative stress and protein and lipid peroxidation. This agent maintained the reduction- oxidation in the cellular of the organs. With the decrease in glutathione followed by an increase in ROS, resulted to subsequent creation of different processes of damage to various organs.

■ **Clinical Manifestation**

● **General**

The first exposure to sulfur mustard is often without pain. People smell only a little garlic or sulfur odor. Naturally, symptoms will be shown for several hours. The maximum severity of symptoms appears after a few days. The most affected organs by contact with sulfur mustard (mustard gas) are eyes, skin and respiratory system (9, 16). It should be noted, that if sulfur mustard is distributed in the air of the places like battlefield, can hurt eyes, skin, and respiratory system. Although, the intensity of injury depends on the dose, route of exposure, protection method, environmental conditions (e.g., temperature and humidity), demographic parameters of the victims (e.g., age, sex, height) etc. (15). The time to start of signs and symptoms is as follows; nausea, vomiting, eye pain in 30-60 minutes, lacrimation, photophobia, rhinorrhea, sneezing, and sore throat in 2-6 hours, erythema, hoarseness, non-productive cough in 6-24 hours, skin blistering, productive cough in 24-48 hours, ocular recovery starts, hyperpigmentation, secondary infections in 2-6 days(21). The lethal oral dose for humans is 200 mg. The amount of 4-5 gr on naked skin in a long exposure time and 1500 mg/min/m^3^ via respiratory system may be the lethal dosage of sulfur mustard(22).

● **Eyes **

The most sensitive organs during exposure to sulfur mustard are eyes. The dose threshold of toxicity of sulfur mustard for eyes and skin are 12 mg min/m^3^and 200mg/min/m^3^, respectively. Even low doses provide incapacitation and visual impairment (23, 24). After an hour of exposure to sulfur mustard, symptoms start with a sensation of grittiness, advance soreness, and bloodshot eyes before edema and acute conjunctivitis. When concentrations are less than 50 mg/min/m^3^, simple conjunctivitis and corneal swelling may appear, and edema occurs when the dose is higher than 200 mg min/m^3^(23, 25). During the 2-6 hour after exposure, patients feel severe ocular pain, laceration, photophobia, blepharospasm, and reduced visual acuity, also temporary blindness after 1 to 12 hours. Usually spontaneous recovery happens after 48 hours, with full regeneration of the corneal epithelium occurring on day 4 to 5, however complete recovery may take 6 weeks or more(22, 23, 26). But the findings are delayed and more severe phase leads to irreversible visual impairments and even blindness (this point is controversial). Based on the animal studies (a few weeks) and investigation on human victims (several years), there is a chemical silent period in epithelial corneal defects and corneal neovascularization (NV), thinning and opacity (24, 27). Severe lesions may be associated by a low grade iridocyclitis without synechia or cataract formation, also intraocular pressure (IOP) may transiently increase for a few days (23, 28, 29). War victims suffered chronic or delayed- onset mustard gas keratitis (MGK) (30). Generally, the victims with MGK usually suffer chronic keratitis, impaired corneal sensation, recurrent/ persistent corneal erosions, limbal stem cell deficiency (LSCD) , corneal neovascularization, lipid and amyloid deposition, irregularity, and corneal thinning and scarring (15, 23). Limbal stem cell deficiency can be mild to moderate. LSCD may cuse distructive loss of limbal epithelial stem cells, or dysfunctional limbal stroma (31). Patterns of eye injuries are posed after exposure to sulfur mustard with the present dose in mg per minute in cubic meter and time duration in table 1. 

● **Respiratory**

After ophthalmologic lesions of sulfur mustard exposure, respiratory symptoms are seen. These complications happen before skin lesions appear (33). Upper and lower respiratory system may be affected by sulfur mustard.(34). While sulfur mustard is being inhaled as spray, mostly the larynx, pharynx and tracheobronchial mucosa are affected. The symptoms appear in the upper respiratory system with pain and discomfort in the nose or sinus, next irritation of nasal mucosa, hoarseness, sneezing and coughing. When exactly developed, symptoms can range from, lacrimation, from not being able to smell or taste, loss of smell and taste, and discharge of mucosal secretions from nose and throat (25, 35). Large volumes of vapor will cause laryngeal injury (aphonia and husky voice and upper medium-sized airway damage (tracheobronchitis) that nonproductive hacking cough usually reveal. The gas in higher concentration can reach into lower parts of the airways and may result in persistent cough, dyspnea, and likely hemorrhage into the alveoli. Finally, the adult respiratory distress syndrome may occur (25). Infection of respiratory system is a common complication after 36-48 hours. Prolonged recovery after 1 to 2 months may occur, especially following secondary infections and necrotic bronchopneumonia(22).

**Table1:** Patterns of ocular damage as a result of sulfur mustard exposure (22, 32)

**Ocular disorders**		**Phase**	**Severity**	**Dose in mg/min/m** ^3^ ** (environment air)**	**Duration**
**Symptoms**	**Signs**				
Foreign body sensation, tearing, photophobia, blepharospasm,	Eyelids hyperemia, vascular dilation and hyperemia of the conjuctive,	Acute	Mild	12-70 ( In some cases, more than 100 to 200)	Up to 2 weeks
Same as mild damage, dry eye sensation, eye pain	Same as mild damage, conjunctival edema, corneal epithelial edema, corneal epithelial erosion, superficial punctuate keratopathy (more in the lid fissure area)		Moderate		
Same as mild and moderate, severe ocular pain, swelling, redness, sores and spasms of the eyelids, reduced vision	Same as mild and moderate, inflammation, edema and in some cases, secondary infection of the conjunctive, ischemia and necrosis of the conjunctive, limbal ischemia and necrosis, corneal epithelial irregularity and defect, corneal stromal edema, possible corneal infection, inflammation of the anterior chamber (uveitis), perforation of the cornea		Severe		
Photophobia, burning, foreign body sensation in eyes, dry eye, tearing, slight redness of the eye	Meibomian gland dysfunction, chronic blepharitis, reduced thickness of the tear meniscus layer, telangiectasia of the conjunctival blood vessels, comma shape vascular tortuosity in the palpebral fissure area (nasal and temporal), subjunctival fibrosis, subconjunctival hemorrage, scarring of the conjunctiva, punctuate epithelia erosions	Chronic and delayed	Mild	100-200 ( In some cases, more than 200)	3-6 weeks
		
Same as mild damage, reduced vision, marked red eye, itchy eyes, ocular pain	Same as mild damage, corneal irregular astigmatism, periods of relapse and remission, mild to moderate limbal ischemia, irregular cornea, thinning of corneal periphery, corneal opacity as well as lipid and amyloid material and deposition in the corneal periphery, peripheral corneal vascularization, peripheral stromal scars of the cornea, peripheral intra-corneal hemorrhage, transparency of the corneal center, decreased corneal sensation		Moderate		
Same as mild and moderate, severe photophobia, severe vision loss, severe pain	Same as mild and moderate, severe limbal ischemia, limbal cell deficiency, thinning and opacity of the central and peripheral parts of the cornea, corneal opacity as well as lipid and amyloid deposition in the cornea, central and peripheral corneal vascularization, band keratopathy and scars in the central and peripheral corneal stroma,central and peripheral intra-corneal hemorrhage, corneal conjunctivalization, corneal descemetocele, corneal ulcer, corneal melting and perforation, history of limbal and corneal surgeries		Severe		
Permanent blindness				> 200	*Very rare*

The important complaints of the late finding of upper respiratory tract in sulfur mustard poisoning include shortness of breath, cough and sputum, intermittent and continuous dysphonia. Laryngoscopic recording expressed inflammation (edema and erythema) in supraglottic and subglottic regions. In general, patients suffered chronic laryngitis. Also, synechia and nodules may have been caused by infectious or chronic laryngobronchitis (36). Chest x-ray assessments of lower respiratory tract indicate obstructive lung disease, hyperinflation, air trapping, increased marking around the bronchioles, and bronchiectasis. In addition, pulmonary function test (PFT) shows decreased forced expiratory volume in one second (FEV_1_), forced vital capacity (FVC), and the ratio between these two volumes (FEV_1_/ FVC) is an indication of obstructive pattern (37). The involvement of pulmonary lesions by spirometry and severity (mild, moderate, severe, respectively) are as follows; 65≤FEV1<80 or 65≤FVC<80 (mild), 50≤FEV1<65 or 50≤FVC<65 (moderate), 40≤FEV1<50 or 40≤FVC<50 (severe)(37). Exposure to sulfur mustard can cause cancer of the upper respiratory system, also some evidence shows that it can result to lung cancer (38).

Generally, respiratory sequelae includes chronic bronchitis, emphysema, tracheobronchomalacia, and bronchiolitis obliterans(16). Several studies confirmed that chronic bronchitis is common late complication by exposure to sulfur mustard in the lower respiratory tract. However, the presence of hypoxemia and hypercapnia from asthmatic bronchitis leads to chronic obstructive pulmonary disease (COPD), cor pulmonale, and respiratory failure in the end stages of the disease (33, 39, 40). High- resolution computed tomography (HRCT) technique confirmed air trapping, bronchiectasis with dyspnea, productive cough and hemoptysis, pleural thickening with hemoptysis and chronic bronchitis (41, 42).

● **Skin**

Skin is an important vulnerable tissue to sulfur mustard. Various factors such as temperature, humidity and anatomical position determine the type of injuries and intensity of symptoms. Sulfur mustard reacts with skin proteins and degrades the cell`s proteins and underlying extracellular matrix (35, 43). After exposure, the cutaneous effects start from 2 to 24 hours. The first signs and symptoms include erythema, skin lesions with or without blister formation, itching and burning sensation that could be seen. Also, large flaccid bullae may progress, then unify, following slough like large sheets of epithelium (Nikolsky’s sign). If large areas are involved with disturbance of water and electrolyte, secondary infection may occur (21, 26, 44, 45). They are usually localized in warm moist areas such as the groin and axilla. Lesions tend to heal slowly, and it frequently causes wounds and blisters (43). Blisters start with small vesicles within erythema. They gradually unify to pendulous blisters with large volumes of clear yellow fluid(25).Hyperpigmentation usually follows after erythema. When melanocyte destruction occurs there will be hypopigmentation(44). 

Acute sulfur mustard exposure on human skin depends on dose and its dosage form; itching, dry and pale of the exposed area (vapor:50-100 mg/min/ m^3^, liquid: 10-20 µg/cm^2^) (46), erythema can often be observed at a threshold dose (vapor: 100-300 mg /min/m^3^, liquid: 10-20 µg/cm^2^) , blister formation occurs at higher doses (vapor: 1000-2000 mg /min/ m^3^, liquid: 40-100 µg/cm^2^) (35). Also, in 50% of people exposed to sulfur mustard in skin with vapor ~ 10,000 mg /min/m^3^, and liquid 100 mg/kg resulted in death (27, 46).

But chronic and delay complications of the skin caused by exposed to sulfur mustard depends on the incidence and insistence of lesions following sulfur mustard exposure. It is directly related to time and intensity of exposure (22). As mentioned, about 80% of the sulfur mustard in contact with skin evaporates, and only 20% of the remaining penetrate into the tissue, namely keratinocytes and hair follicle cell membrane. It cannot be removed in ten minutes, and will bind to the epidermal and dermal tissue, often in the cornified layer (47, 48). 

In most studies, the patient’s complaint of itching followed burning sensation and desquamation, due to dryness of the skin, especially in dry weather and physical activity. Axilla, scrotum, and anal region have high humidity and sensitivity to the exposure (49). Sulfur mustard vapor results in 1^st^ or 2^nd^ degree burns, and its liquid in full thickness burns, because it easily penetrates normal military uniforms. Therefore, it causes gluteal, perineal, and scrotal burns. Mild burns usually heal spontaneously and with ordinary care. However, deep burns are a candidate for skin graft (16, 48).

Other complications noted in late skin lesions are excessive dry skin (xerosis), itching, hyperpigmentation and hypopigmentation, local hair loss, eczema, chronic urticarial, and cherry angioma (25, 33, 47). When blister erupted, a necrotic layer or eschar is formed on the skin. The wounds usually heal over the period of 10-50 days, pigmentary changes may persistently remained for months or years(19). Pigmentation can decrease or increase in function at late skin disorder in the location of primary sulfur mustard lesions. If melanocytes are healthy, hyperpigmentation occur. However, the effects of sulfur mustard on the pigmentation can appear as hypopigmentation. In the case of melanocytes are destruction, depigmentation well be diagnosed (25, 37, 50). Another point is the appearance of cherry angioma and telengiectasis, which is seen in patients exposed to sulfur mustard. Cherry angioma is a benign vascular neoplasm (50). 

● **Mutagenicity, Teratogenicity, and Carcinogenicity**

There is no evidence for the mutagenicity of sulfur mustard and no document of teratogenicity was found in rats with different doses of sulfur mustard (51).Sulfur mustard is an alkylating agent that affects DNA. It may induce long-term cancer after exposure (52).Sulfur mustard is classified as a carcinogen by the international agency for research on cancer (IARC).Human studies indicate an association between occupational or battlefield exposure to sulfur mustard and induce respiratory, skin, gastric cancers. There are many reports of leukaemia and upper respiratory tract malignancies in old Japanese, British and American workers of factories that manufacture sulfur mustard. Lung cancer, nasopharanx and bronchogenic carcinoma, adenocarcinoma of stomach, as well as acute myeloblastic and lymphoblastic leukaemia have neen reported among the Iranian veterans (21, 22, 51, 52).

●**Hematopoietic andimmune and systems**

Leukocytosis is usual in the first few days after sulfur mustard exposure. White blood cells (WBCs) count begin to decline on the 3^rd^ and 4^th^ days after exposure and reach their minimum level around the 9^th^ day. Lymphocytes are the first line to disappear and granulocytes are also strictly affected but they are reduced with delay after lymphocytes (51). Leukopenia reaches a lowest count about 10 the day after the exposure (21). Thrombocytopenia and anemia appear later (53). Bone marrow biopsy demonstrates a decrease in cell numbers and cellular atrophy. High dose exposure induces a cytotoxic effect in hematopoietic stem cell leading to pancytopenia (53).

Immune responses are categorized into two types: humoral, which is intervened by antibodies and cellular which is mediated by T cells (54). Sulfur mustard poisoning can impair both humoral and cellular immune functions (51). Sulfur mustard may increase the levels of IgG and IgM during the first week, but their levels decrease over the next 6 months (55, 56). Sulfur mustard is also effective on complement system (57). Complement changes are likely related to the acute phase response following infections, and may indicate the efficiency of the classic pathway in the complement system (58). Both C_3_ and C_4_ levels increase, then a gradual decrease is seen over one year, remaining up to three years after exposure, specifically in patients with severe poisoning (51). T helper cells remarkably decreased while T suppressors increased in patients exposed to sulfur mustard(58). Also, check out on CD_45_ (common leukocyte antigen present on 99% of leukocytes), CD_56_ (natural killer (NK) cell marker present on 70% of NK cells) and CD_25_ (interleukin_2_ R (IL) present on activated NK cells) marker (54, 57). Most studies revealed that there is a probability of impairment in cellular immunity especially NK cells by sulfur mustard. In people exposed to sulfur mustard there are risks of cancer and also recurrent fungal and viral infections(57). In addition, the assessment of the ratio of CD_45+_ /CD_56+_ cells, CD_56+_ /CD_25+_ cells, CD_8+ _/ CD_56+_ cells that are importantly lower, and noticeably higher, within the normal range in severe patients exposed to sulfur mustard was compared with the control group, respectively(54).

● **Endocrine and Reproductive systems**

Azizi et al. (2001) reported the effect of sulfur mustard on endocrine systems and showed a decrease of thyroid hormones and an increase in reverse T_3_ (rT_3_). The following has reported an increase in adrenocorticotropic hormone (ACTH) during first week after exposure, increase in free plasma thyroxine index (FT_4_I), a thyroid stimulating hormone (TSH) after three weeks of exposure, a continuous increase of ACTH up to week 5, and a significant reduction in cortisol in weeks 4 and 5 after sulfur mustard poisoning(59). Safarinejad (2001) found that the total and free testosterone and dehydroepiandrosterone (DHEA) levels noticeably decreased in the first 5 weeks after exposure. Follic- stimulating hormone (FSH), luteinizing homone (LH), prolactin, and 17 alpha-OH progesterone were normal in the first week. LH increased in the third week while FSH and prolactin increased in the fifth week. All hormone levels returned to normal in the twelfth week after exposure (60). Marzony et al. (2016) reported that sulfur mustard caused wide changes of structural and functional defects in reproductive system including disturbances in the levels of reproductive hormones, testicular damages, sexual dysfunction, genital lesions, impaired spermatogenesis, poor sperm quality (count, motility, morphology, viscosity, volume) and reduced fertility(61).Several studies on testicular biopsies revealed partial or complete stop of spermatogenesis, atrophy of the germinal epithelium, intact Sertoli cells, and normal appearance of Leydig cells (60, 61).

● **Other Systems**

Gastrointestinal (GI) tract could be affected following sulfur mustard exposure. The most common GI symptoms are as follows: nausea, vomiting, anorexia, abdominal pain and diarrhea in the first 24 hours. In some victims, acute gastroduodenitis with hemorrhagic erosions, acute desquamative enteritis, and severe hemorrhagic necrotic colitis have been reported (22, 51). Pancreatic autopsy findings were chronic inflammation, fibrosis, duct ectasia and acinar atrophy (62).

Convulsion, dizziness, anorexia, vomiting, and increased cholinergic activity may be observed following central nervous system acute toxicity of sulfur mustard. Chronic toxicity includes debility, decreased vitality, attention deficit, increased sensibility, impotence, and cardiac autonomic abnormalities (63). Headache, anxiety, fear of the future, restlessness, confusion, and lethargy are seen in mild and nonspecific neurological manifestations (11). Delayed neuropathic disorders were seen in peripheral nervous system (11, 51). Some victims had pure sensory polyneuropathy and the other had sensory-motor distal polyneuropathy of axonal type. Sensory nerve impairments comprise hyperesthesia, hypoesthesia (sign), and paraesthesia (symptom) that were the most ordinary clinical complications (64). 

Balali-Mood et al. (2005) reported abnormalities in the peripheral nervous system as results of electromyography (EMG) and nerve conduction velocity (NCV) Iranian veterans. They showed sensory nerve disorders more than motor nervous disorders, as well as, the prevalence of these problems were in lower extremities more than the upper extremities. EMG was normal in some patients, whereas in the other patients had incomplete involvement with normal amplitude, and the rest of patients incomplete involvement with low amplitude(11). NCV and EMG disturbances in both the upper and lower extremities are frequently treated as symmetric(25). In fact, the available documents posed long term axonal neuropathy in these patients(63).

The symptoms and complications of sulfur mustard poisoning and cardiovascular system were chest pain and palpitation which were the most frequent symptoms and hypertension was the most common complication (49). In electrocardiography (ECG) findings, there are no heart abnormalities among the sulfur mustard exposed victims in the acute phase in hospitals, but Karbasi- Afshar et al. (2017) reported some disturbances in the exercise test and echocardiography (65). Moreover, the incidence of coronary atherosclerotic lesions among these patients was significantly higher than the control group; although, the type of lesions was not different (66). Shabestari et al (2011) suggested coronary artery ectasia (CAE), a late toxic effect of sulfur mustard in veterans. The prevalence of coronary artery ectasia in these veterans was 7.5 times more than non-exposed individuals. Besides the most generally involved artery in these victims was the left anterior descending (LAD) artery (67). Cardiac dysrhythmias occur in the indication exposure to high doses of sulfur mustard (68). In these victims, it seem that coronary artery diseases, especially coronary ectasia, and ventricular dysfunction can cause noticeable cardiovascular abnormalities(65).

■**Laboratory Diagnostic Tests**

Sulfur mustard urinary metabolites are appropriate for detecting its contamination, but the most difficult diagnosis is the rapid elimination of sulfur mustard. Protein macromolecular adults could play an important role as long- term biologic markers of sulfur mustard exposure (22, 69). There are four main metabolic pathways and four types of sulfur mustard biomarkers in urine, blood and blister exudates, as well. The first pathway involves the direct oxidation product of sulfur mustard, bis-ß- chloroethyl sulfoxide (SMO), the directly hydrolyzed metabolite thiodiglycol (TDG), and its oxidation product thiodiglycol sulfoxide (TDGO). 

The second pathway involves a reaction with numerous glutathione, then can undergo oxidation change to the sulfone by ß-lyase cleavage. It can lead to formation of 1, 1’- sulfonylbis [2-S-(N-acetylcysteinyl) ethanol] ((SBSNAE), 1, 1’- sulfonylbis [2- (methylthio) ethane] (SBMTE), 1-methylsulfinyl-2- [2-(methylthio) ethylsulfonyl] ethane (MSMTESE) and 1, 1^’^- sulfonylbis [2-(methylsulfinyl) ethane] (SBMSE). 

The third pathway is the reaction on the certain nucleophilic sites in DNA to produce SM-DNA adducts. The main sites of DNA alkylation by sulfur mustard include N^7^, O^6^ positions of guanine, N^3^ position of adenine, and interstrand or intrastrand crosslinks at the N^7^ position of guanine, and adducts of N^7^-[2-[(2-hydroxyethyl) thio] ethyl]-guanine (N^7^-HETEG), O^6^ -[2-[2-hydroxyethyl) thio] ethyl]- guanine (O^6^-HETEG), N^3^- [2-[(2-hydroxyethyl) thio] ethyl]- adenine (N^3^-HETEA), and bis [2-(guanine-7-yl) ethyl] sulfide (Bis-G). The fourth pathway involves the reaction with different amino acid residues present in proteins, among which are the HETE-valine (HETE-Val) adduct of hemoglobin and HETE-cysteine adduct of albumin (69, 70). 

● **Diagnosis of urinary metabolites of sulfur mustard**

The urine samples are collected to determine free and conjugated forms of the simple hydrolysis product thiodiglycol (TDG) and TDG sulfoxide (TDGO) [its oxidized form]. Free TDG, free plus conjugated TDG (total TDG), free TDG+ TDGO, and free plus conjugated TDG + TDGO (total TDG + TDGO) could be evaluated. Liquid chromatography- mass spectrometry (LC-MS) and gas chromatography- mass spectrometry (GC-MS) methods are carried out to analyze the samples(69, 71). TDG and TDGO have low concentrations in human urine. The measurable amount of total TDG + TDGO excreted in urine during the first five days accounted for 0.5-1 % of the practical dose of sulfur mustard. Therefore, this diagnostic method will be useful for a short term (11, 71).

GC-MS and GC-MS-MS (detection limits lower than 0.1 ng/ml), were developed for the analysis of the ß- lyase metabolites in urine of the victims (72). Nowadays, rapid method is introduced to analyze the ß- lyase metabolitis in urine using LC-MS-MS with electrospray ionization (ESI) detector. LC-MS-MS provides an alternative to GC-MS-MS, to avoid the conversion of the metabolites to a less polar and more volatile analyte (73).

● **Diagnosis of sulfur mustard adducts with DNA**

DNA and protein adducts have partly longer times from week(s) to months and can be used as good biomarkers for analysis. DNA has large affinity towards alkylating agents, which is the exact reason for the cytotoxicity of sulfur mustard. The main site of actions is the N^7^, O^6^ position of guanine and N^3^ position of adenine about DNAs to be alkylated by sulfur mustard. As explained, there are four kinds of sulfur mustard-DNA adducts, for recognizing and using biomarkers, i.e., N^7^-HETEG, Bis-G, N^3^-HETEA, O^6^-HETEA.Based on an in vitro study, in calf thymus DNA or human blood, N^7^-HETEG has the most quantity (61%), Bis-G (16%), N^3^-HETEA (11%), O^6^- HETEG has the minimum amount (0.1%) of the total sulfur mustard-DNA. However, O^6^- HETEG has the minimum percentage, but it is the main responsibility of DNA damaged by sulfur mustard (70). The enzyme - linked immunosorbent assay (ELISA) is applied for detecting DNA-sulfur mustard adducts (74).

● **Diagnosis of Sulfur Mustard adducts with proteins**

Because the metabolites cannot be detected in urine for a long time, therefore protein adducts to blood are a potential tool to assess exposure. Hemoglobin and albumin are two numerous proteins in blood that can be simply separated to determine sulfur mustard adducts(11). A number of adducts (histidine residues, glutamic acid residues, and both of the N-terminal values) N_1_ and N_3_ histidine adducts were found to be most abundant, and it was the alkylated N- terminal valine adducts that were most useful for next measurement (75). Sulfur mustard forms adducts to hemoglobin at valine, glutamic, and histidine residues can be available around 120 days. Furthermore, sulfur mustard can form stable adducts to human serum albumin (HAS) at its reactive cystein-34 residue. Its half-life is shorter than hemoglobin, and is 20 to 25 days. Sulfur mustard binds to the single reaction cystein residue of human serum albumin, which contains a stable-hydroxyethylthioethyl [S-HETE] adduct. S-HETE adduct can be used as a long-term biomarkers of sulfur mustard exposure in humans. It can be measured using LC-MS-MS (76). 

■ **Treatment**

● **First aid measures and triage**

Victims should be transferred to a safe area as soon as possible. All victims’ clothes should be taken off and discarded. The skin should be rinsed with tap water and neutral soap (pH near 7). Rubbing and dry cleaning the skin may increase penetration of sulfur mustard into bloodstream. Whenever contamination occurs in the area affected with liquid mustard, eyes should be washed with large volumes of water, normal saline or ringer solution. Then the victims should be transferred to a medical center or hospital (77, 78).

After chemical warfare agents (CWAs) release, a triage program should be performed in the clean area (warm zone) to determine the priorities for resuscitation, decontamination, pharmacological therapy, and transport to hospital. Triage is a dynamic process and should be carried out continuously in both contaminated (hot zone) and clean zones. The triage programs include; T_1_ (immediate or urgent): victims who need medical care and advanced life support within a short time on the event location and in the hospital. T_2_ (delayed) victims with injuries who are in need of prolonged care and require hospitalization, but delay of this care does not affect the prognosis of the event. T_3_ (minimal): victims who have minor injuries who will not be evacuated and will be able to return to duty in a short time. T_4_ (expectant): victims with fatal injuries who will probably not survive in the medical care available before reaching terminal care (78). According to the colors and seriousness of exposure at the battlefield, red, yellow, green, and black colors were set for immediate or urgent, delayed, minimal, and expectant classes (79).

● **Medical treatment **

■ 1) **General treatment and suggested antidotal treatment**

In clinical conditions, it is possible to use sedative to control the patient’s pain and induce relaxation (22). At present, there is no proven evidence of clinical and therapeutic effects on the use of extracorporal detoxification procedures, such as hemoperfusion and hemodialysis (22). There are no recognized antidotes by official sources for sulfur mustard. Although based on studies on laboratory animals to protect the toxic side effects of alkylating agents, sodium thiosulfate has been introduced as its antidote. But there are no supplementary clinical reports to confirm it and it is not recommended to use in intoxicated persons (5).

■ **2) Special systems’ care**

◘ **2-1)** **Skin lesions management**

The treatment plans are based on reducing the risk of acute and chronic sequelaes (5). After the victims contaminated clothes being gently removed, the historical advice is to rinse exposed skin with tap water and neutral (pH of near 7.0) soap. Some experts believe that the skin could be washed with home bleach solution or hypochloride 0.5% solution. Other researchers considered that the skin was washed with chloramines -T 0.2%- 0.3% solution at least six times a day (13, 77). A wide variety of solutions is presented in the theoretical discussion of this problem, including water, normal saline, sodium bicarbonate 1.5% solution, saturated sodium sulfate or magnesium sulfate solutions (hypertonic solutions), boric acid solution, dichloramine -T 0.5% solution in a solvent, and dilute solutions of sodium hypochlorite or potassium permanganate solution 1/10000 and warm water. The important point is that there are no studies in either laboratory animals or humans systematically, which put forward the benefits of any therapeutic approach, compare them, and prioritize each of them (5). 

At first, the skin is pale and then it becomes erythematous after several hours. Blisters do not usually appear until the second day, and develop for a few days. Therefore, for areas of erythema and small blisters (≤ 2 cm) there is the use of soft lotions such as calamine and local steroid solutions. This action reduces itching and irritation (22, 80). Besides, the use of topical bacteriostatic agents, such as silver sulphadiazine 1% (Flamazine) could prevent secondary infections. One of the common problems is the moderate pain and itching, and it is possible to use mild analgesics, antihistamines and low doses of diazepam. In severe pain there is a need to use narcotics such as morphine sulfate (80). According to a study, Panahi et al. compared the combination of phenol1% and menthol 1% to relieve itching and other skin lesions with the placebo group. They found significant differences before and after treatment with the mentioned drugs. As a result, they recommended the combination of phenol1% and menthol1% in the treatment of chronic skin lesions due to sulfur mustard exposure (81). Panahi et al. compared the effect of pimecrolimus cream 1% (an immunosuppressant which inhibits calcineurin in skin) and betamethasone cream 0.1%.in chronic skin lesions after sulfur mustard exposure. This study showed that the effect of pimecrolimus cream 1% is better than betamethasone cream 0.1% in the control of pruritus, burning sensation and dry skin, specialty in the thorax, back and upper extremities (82). To eradicate chronic skin lesions, another study has been performed by Panahi et al. They used doxepin cream 5% or betamethasone cream 0.1% twice daily for 6 weeks. Both groups showed significant progress regarding pruritus, burning sensation, skin dryness, and skin scaling. The lesions of all areas significantly reduced after treatments, except on the head, face, and genitalia. This study posed the same efficacy between doxepin cream 5% and betamethasone cream 0.1% .Therefore, doxepin 5% is a potential alternative to control pruritus and other skin lesions (83). At last, in a study by Shohrati et al, they used oral drugs such as cetirizine 10 mg, doxepine 10 mg, hydroxyzine 25mg daily for 4 weeks. Hydroxyzine 25 mg once daily has equal result in comparison to doxepine 10 mg once daily, but more than cetirizine 10 mg once daily in the control of chronic pruritus in these patients (84).

In patients with delayed cutaneous complications of sulfur mustard exposure (DCCS), has shown that capsaicin (0.025% as cream) decreased the scaling of pruritus and skin dryness less than betamethasone (0.1% cream), but the burning sensation in capsaicin-treated group was higher than the control (85).

After these measures, the therapeutic approach with blisters is as follows; the small blister (≤ 2 cm) will remain intact and does not get debridement. However, they have ruptured spontaneously. Debrideinent may help to increase healing process. In the blister (> 2cm), the liquid is evacuated by syringe or incision, then the debridement is done, washed with normal saline and dressed with silver sulfadiazine (11, 13, 77). Sulfur mustard causes large scale damage to the skin from superficial to deep dermal, as well as full thickness burn in humans. This injury depends on damage to the DNA. This point may delay or prevent the effective replication of the keratinocytes. Failure to replication causes long healing process, it means, confronted with damage dermal , also a lack of a favorable matrix on the new epidermal (86, 87). A term called “dermabrasion” means to remove the necrotic surface from the burn area, active regeneration of new epidermis from viable epithelium at the edge of the wound. In addition, debridement with laser (lasablation) facilitates wound healing at the cellular level, and is done in different ways; Powered dermabrasion, pulsed CO2 laser ablation, and Erbium: yttrium-aluminum-grant (Erb: YAG) laser ablation could accelerate the rate of healing of full thickness skin. In severe and extensive burn, skin graft may be required (48, 86, 87).

◘ **2-2)** **Management of the respiratory toxic effects**

Nature of sulfur mustard induces lung damage and the word “mustard lung” is used to support specific entity. The best time to start treatment interventions in acute phase is when the clinical signs have been seen. After the acute phase, long term disability is the greatest problem in this system for people exposed to sulfur mustard (88).

● **Physiotherapy & Oxygen therapy**

The foundations of the treatment in these patients are respiratory physiotherapy, oxygen therapy, antibiotics, and mechanical ventilation (89). Respiratory rehabilitation plays an important role in the medical treatment of patient with sulfur mustard. Respiratory physiotherapy included postural drainage and chest percussion and vibration (90). Long term supplemental oxygen therapy and nasal intermittent positive pressure ventilation has been ordered (88). 

Heliox, a mixture of helium: oxygen (79:^21^) instead of air: oxygen (79:^21^) with non-invasive positive pressure ventilation (NIPPV) can be used in constricted airways with less turbulence. This method decreases dyspnea and work of breathing, reduces intrinsic positive end expiratory pressure and dynamic hyperinflation. Moreover, it had beneficial effects on systolic, diastolic and mean arterial pressure, pulse rate, respiratory rate and dyspnea, also higher arterial oxygen saturation (85, 91). 

●**Antibiotics (Macrolide Antibiotics)**

Sulfur mustard inhalation causes inflammatory responses, followed by respiratory dysfunction. One of these disorders is secondary infections. Antibiotics are recommended to decrease or eradicate secondary infections. In patients with bronchiolitis, because of no response to full dose corticosteroids, prednisone and azithromycin may be helpful. Moreover, a combination of clarithromycin and actylcysteine for 6 months was effective in chronic bronchitis and bronchiolitis (90, 92).

Macrolide antibiotics have shown effectiveness in different chronic respiratory diseases such as diffuse panbronchiolitis (DPB), asthma, cystic fibrosis, chronic bronchitis, and chronic sinusitis (92). Macrolides have anti-inflammatory and immunomodulatory effects and the famous macrolides are; erythromycin, clarithromycin, roxithromycin, azithromycin, and josamycin. Macrolides can have reduced airway inflammation with various mechanisms. The important point is the lack of eosinophilic inflammation (neutrophil mediated inflammation). In non-eosinophilic inflammation, macrolides are the best choice among antibiotics to play anti-inflammatory role. These mechanisms include reduced airway mucus secretion, and anti- inflammatory properties including decreased airway neutrophil collection in the expression of pro-inflammatory cytokines, e.g., interleukin (IL-6,IL-8), also CRP, RF and the increase expression of markers of inflammation which include cyclooxygenase-2 (COX-2), tumor necrosis factor alpha (TNF α), inducible nitric oxide synthase (iNOS), matrix metalloproteinase-9 (MMP-9) and a notable increase in total protein, IL-1α and IL-13 (88, 93).

●**Bronchodilators**

Bronchodilators can be used in patients with enhanced airway hypersensitivity, plus people with moderate to severe lung injury due to sulfur mustard exposure. The combination of ß- agonist (e.g., salbutamol, trade name; albuterol, ventolin) and an anticholinergic (e.g., iprotropium bromide, trade name; atrovent) is more effective than any other bronchodilators if used alone(94).Bronchodilators can reverse signs and symptoms in asthma and airway obstruction. They cause to improve characteristics of pulmonary function tests (PFTs). Prescribing 200 µg of salbutamol is remarkably inhalable to improve PFT in these patients. Furthermore, they undertake to decrease pulmonary complications such as severe bronchial stenosis and loss of ciliary movement that is directly related to chronic infections and bronchiectasis. In addition, some victims suffered obstructive airway disease after long- term exposure, bronchodilators could decrease or resolve it (95).

●**Corticosteriods**

Corticosteriods are extensively used to resolve respiratory signs and symptoms of mustard lung. Of course, the use or non-use corticosteroids in these patients is discussed and being controversial. However, some studies have shown that they are used to manage severe chronic bronchitis like most asthmatic patients. It is noteworthy that inhaled corticosteroids can progress pulmonary function in these patients, and this has a synergistic effects with inhaled ß-2 agonist bronchodilators (96, 97). In patients exposed to sulfur mustard suffering from chronic bronchiolitis, a combination of inhaled corticosteroids, long- acting ß-2 agonists is recommended. Moreover, the medium dose of fluticasone/ salmeterol would have a similar effect as taking high doses of beclomethasone with short-term beta-agonist for airway reversibility (96, 98).

●**N-acetylcysteine (NAC)**

Long term use of corticosteroids causes adrenal suppression, osteoporosis, and sodium retention. In parallel with this point, patients who do not respond well to bronchodilators, based on a series of researches N-acetylcysteine (NAC) can be helpful. NAC that is a thiol compound, and chemical formula C_5_H_9_NO_3_S can be an appropriate substitute instead of old and traditional treatments (99, 100).

People exposed to sulfur mustard have structural changes in their lungs. These changes cause irreversible injury of parenchyma and airway walls. In these patients, inflammation and oxidative stress play a main role in the phathogenesis of many disorders in respiratory systems (101). Glutathione (GSH) is the principle thiol contribute in cellular redox reactions. It is involved in the detoxification of most endogenous and exogenous toxic substances. GSH is released in cellular response to xenobiotics, free radicals, reaction oxygen species (ROS), and other origins of cellular damage (102). In fact, available data in vitro and in vivo showed that NAC protects the lungs against toxic agents, in two ways; first, the increase of pulmonary defense mechanism with direct antioxidant properties, second, is the indirect role as a precursor of GSH synthesis (101). Bobb et al. (2005) and also many other researchers claimed; N-acetyl-L-cysteine (NAC) is a candidate chemoprophylaxis substance for sulfur mustard exposure in humans, in general, it is has a clinical application (102).There are many studies that confirmed the helpful effects of NAC in humans such as; preserve oxidant- antioxidant homeostasis due to increasing GSH, decrease in the amount and the activity of inflammatory cells and ROS production, prevent the release of several inflammatory mediators in various pathological situations, decrease the secretion of several inflammatory modulators, and reduces product ROS (99, 103).

NAC administered orally a maximum dose of 600 mg/daily, or 1200 mg/daily, 1800 mg/daily, confirmed the useful effect of N-acetylcysteine on intensity scale. High dose NAC for high risk patients with severe conditions and or those patients who are still in a moderate phase of the disease (103). The authors of this article believe that many studies require high dose (1200 mg/ daily and 1800 mg/ daily) of NAC that is more effective than 600 mg or not. Now, some researchers and experts accept this subject and others reject it. Based on approved studies, therapy of NAC may improve the lung function.

**● Recombinant tissue plasminogen activator (rt-PA)**

The FDA has approved recombinant tissue plasminogen activator (rt-PA) in long-term chronic sequelae of sulfur mustard inhalation exposure, such as bronchiolitis obliterans (BO) and pulmonary fibrosis. It is directly delivered to the airway using bronchoscopy(104).

●**Interferon gamma-1b (INFγ- 1b)**

Exposure to sulfur mustard or mustard gas causes disorders such as inflammation respiratory system. Transforming growth factor ß1 (TGF- ß1), an isoform TGF-ß, plays an essential role in some of the pathogenesis of respiratory system. TGF-ß, specially TGF- ß1 is enhanced in patients exposed to sulfur mustard. Also, IFN-γ 1b is a bioengineered form of interferon gamma. IFN-γ had anti-inflammatory effect via downregulation of TGF- ß and type land III procollagen gene expressions. As level of TGF- ß increased, IFN-γ may be effective in pathologic situation. Interferon gamma-1b (200 µg) three times per week subcutaneously and low dose prednisolone (7.5 mg) once a day orally for six months could improve dyspnea indices and pulmonary function tests, a decrease in hospitalization time, an increase in arterial oxygenation of sulfur mustard exposed with severe delayed lung complications(88, 90, 105, 106).

●**Sildenafil and Tadalafil**

Sulfur mustard poisoning can lead to pulmonary arterial hypertension (PAH) (107). This disease is determined by proliferation and regeneration in these vessels. PAH increases in pulmonary vascular resistance (PVR) and finally induces right ventricular failure and death(108). Recently, several new drugs in phosphodiesterase -5 (PDE5) inhibitors have been approved by Food and Drug Administration (FDA) for the treatment of pulmonary arterial hypertension. PDE5 inhibitors increase cellular cGMP in vascular smooth muscles. This action will cause vasodilation [e.g., pulmonary artery smooth muscle cells (PASMCs] and decreases pulmonary vascular resistance (PVR), pulmonary vascular resistance index (PVRI), PVR/SVR ratio and pulmonary arterial pressure (PAP), also cardiac output (CO) increase. One of these drugs introduced in 2005 is the sildenafil (107, 109, 110).

Sildenafil would be in a starting dose of 12.5 mg three times daily. Then the dose is gently increased to 150 mg/day every 12 weeks. The optimal dose of sildenafil for PAH is not certain. Although this dose may be various in clinical managements, satisfactory effects have been reported over to 500 mg/day (110). On the basis of studies, sildenafil is a better selection, because it is effective, simply accessible, relatively cheap, simple to use, and very well-tolerated without any main side effect. It may be the first choice drug for PAH patients (110, 111). 

**Tadalafil**

According to tadalafil’s structure, it has different pharmacokinetic properties and longer half-life compared to sildenafil (the terminal half-life of sildenafil is 3-5 h). It is assigned in the treatment of pulmonary arterial hypertension. Tadalafil is prescribed 2.5 mg, 10 mg, 20 mg, or 40 mg daily for 16 weeks (108).

●**New Therapeutic approach & Herbal therapy**

Using hypertonic solutions, such as mannitol and hypertonic saline have several effects on the respiratory system. There are numerous studies that mentioned the significant effects of these agents on this system, including the most important; developing an osmotic gradient as the flow of water is pushed towards the airway lumen, which caused reduced viscoelasticity, surface tension, contact angle and sputum content. Therefore, inhaled mannitol is a suitable alternative as anti- inflammatory therapy in COPD, avoid using inhaled steroid treatment, and prevent overtreatment of COPD. Besides other effects are the increased mucus hydration and mucociliary clearance is reduced. also reduced airway wall edema. Nebulization with 5% hypertonic saline proved which one performs that is simple, cheap, easily applied, safe, and seemingly an effective treatment could be generalized for use in clinics, infirmaries, army and general hospitals. It may be remarkable to current treatment [bronchodilator therapy (albuterol/ salbutamol or epinephrine), corticosteroids and nebulized normal saline]. This method has been able to improve the severity of bronchiolitis, cystic fibrosis and reduces the length of stay in the hospital (112-115).Usually herbal and non-chemical agents (phytomedicine) are used in patients who have poor response to common and conventional chemical drugs as an alternative choice for relieving, obliterating or eradicating (116).

*Thyme* with scientific name “*Thymus Vulgaris*”, is a plant from *Labiatae*. With strong expectorant, reduces bronchospasm, relieves inflammation and coughing properties. Other effects include increased ciliary clearance, pause in cholinergic tone, increased vascular continuity, and cease of the release of chemical intermediates from mast cells and basophils. Dosage of thyme to 15 drops of 2% in glass water after each meal (117).*Zataria multiflora* (ZM) [*Shirazi Avishan*] is from the Labiatae family, a flowering plant similar to t*hyme* (*Thymus vulgaris*). Its leaves are used in herbal medicine. Its extract has antibacterial and anti-inflammatory effects. It is generally used as an antiseptic and antitussive for the treatment of respiratory system disorders. This point is confirmed and recommended in many studies (116, 118). In general, *Thyme extract* is useful in upper respiratory infections. In addition, *Zataria multiflora* (ZM) is used to treat common cold. It has been approved for its efficacy and safety (116). 

Another herbal medicine is the combination of *Thymefluid extract* and *Primrose root tincture* at a dosage of 30 drops (1 ml), receive orally five times a day, which is a safe and well tolerated treatment for patients. It reduces the symptoms of bronchitis and the duration of acute bronchitis is shortened (119, 120). The other combination contains *Ivy leaves*, *Thyme herb*, *Aniseed* and *Marshmallow root*. This herbal syrup seems to reduce the cough from common cold, bronchitis and mucus formation in the respiratory system diseases (121). Combination of two types of herbals, such as, *Thyme herb* (*thyme herba*) with secretolytic, expectorant, bronchospasmolytic, antibacterial and antiphlogistic properties and *Ivy leaves* (5.4 ml three times daily for 11 days) are used. The combination is well-tolerated and seems to be a favorable altenative therapy. There is no risk for the progress of resistant pathogens, when repeatedly used in mild respiratory system infections (122).

*Nigella sativa has* a histamine antagonist and inhibitor of the histamine receptor. Also, it is affirmed as anti- inflammatory, antitussive and anticholinergic (123). Boiled aqueous extract of *Nigella seed* was used on chemical war victims. Extract of *Nigella sativa* improved symptoms and PFT values in these victims(90). *Pelargonium sidoides* extracts are spaciously used in the treatment of respiratory system infections. They have antimicrobial and anti-inflammatory properties. They release tumor necrosis factor α (TNFα), nitric oxide, and increased natural killer (NK) cells activity. Therapeutic dosage is 30 drops three times per day for at least seven days. Moreover, *extract Pelargonium sidoides *is available on the market in the form of 10, 20, 30 mg tablets, which can be used three times a day (123).

One of the herbal drug is *curcumin*. There are so many documents of which prove that *curcumin* has an anti-inflammatory propetions. Generally, it can be a regulated different pro-inflammatory gene in various cells (124). In addition, it has antioxidant and free radical scavenging activity properties because it can prevent membrane lipid peroxidation as well as oxidative DNA damage(124). Therefore, it seems that it can affect on the control of severity of sulfur mustard damage disorder(88). Curcuminoids are phytochemicals with significant anti-inflammatory properties used in patients who were suffering from chronic sulfur mustard- induced pulmonary complications. They are orally adminisered at 500 mg T.I.D for 4 weeks(125). In patients with delayed respiratory complications of sulfur mustard (DRCS), some pulmonologists offer the combination of curcuminoids (1500 mg/day) and piperine (15 mg/day) for 4 weeks. This combination improves FEV1/FVC and may decrease inflammatory mediators, including interleukins 6 and 8, tumor necrosis factor-alpha (TNF-α), transforming growth factor- beta (TGF-β), high-sensitivity C-reactive protein (hs-CRP), calcitonin gene-related peptide (CGRP), substance P and monocyte chemotactic protein-1(MCP-1) (85, 126). Today, phytomedicine has become a part of pharmaceutical market and treatment in Asia and Europe. Herbal medicines have safetiness, efficacy and quality. It is then used alone or with conventional medicines (123). Finally, there is a strong evidence of the use of herbal medicines to control the severity of the disease in patients exposed to sulfur mustard. Of course, all these cases will require more researches with more samples.

● **Admitted to ICU**

In victims with severe injuries of the respiratory system, due to damages occurring in the process of injury, the need for hospitalization in the ICU departments will be more special to monitor the patients and treatments more precisely (22).

◘**2-3)** **Eye lesions management**

**2-3-1)** **General measures and medical treatment**

Eye damage is the most incapacitating effect of military force and civilian people. It has been pointed to cause many term eye problems (5).The eyes of victims must be washed as early as possible at the first 10-15 minutes after exposure even if these victims did not have any symptoms in their eyes. Since sulfur mustard induces rapid and irreversible reactions with eye tissues, irrigation technique of eyes is not effective after this time (11).

Too much irrigation with water, normal saline, sodium bicarbonate 1% or 1.5%, dichloramine –T 0.5%, saturated solution of boric acid, liquid albolene, dilute solution of sodium hypochlorite or potassium permanganate and olive oil. There are no proven studies in animals or humans among all these solutions that are more effective than tap water (5, 11, 89). Meanwhile, pads, gauzes and bandages must not be used for the eyes, because they cause worse effects to eyes. This action can lead to raise temperature in the damaged eyes and create lesions (13). In addition, local anesthetic drops should be avoided in both the healthy and injured corneas for ophthalmologic examination (11, 13).

The use of dark sunglasses was offered to victims with photophobia. (13). In general, local steroids must be prevented if there is an evidence of corneal epithelial defects. Although these agents can reduce chemosis and corneal epithelial edema (22).

Mydriatic drops e.g., cyclopentolate and atropine can reduce ocular pain because of spasm of the ciliary muscle and prevent posterior synechiae. Antibiotic drops e.g., sulfacetamide, neomycin, gentamicin, and acidamphenicol, polymyxin-B- sulfate are used to prevent secondary bacterial infections and topical antiglucoma medications to control intraocular pressure (IOP) (31, 35). An anti-inflammatory treatment will be useful for a short period of time after exposure to sulfur mustard (specially at the start of the first hour) for a week or for symptomatic treatment in the formation of corneal neovascularization (NV). Using dexamycin (dexamethasone+neomycin) as an anti-inflammatory agent reduces the symptoms of the eyelid, conjunctiva, and cornea. Clinical observations and specific detection reduce about 50% in severity of corneal injuries following treatment, but dexamycin will have no effect on cornea erosion. By measuring the thickness of the cornea, reducing the thickness and edema, and these agents will also reduce neovascularization. Given these points, the use of dexamycin and other anti-inflammatory drugs is confirmed (24). Matrix Metalloproteinase (MMP) inhibitors, like tetracycline and doxycycline inhibit the activity of MMP with independent mechanisms, in addition to having antimicrobial character. They have anti-inflammatory properties to reduce acute and delayed injuries and reduce the formation of neovascularization in the cornea. The potential value of tetracycline in the treatment of moderate to severe eye injury in the past has been shown. The mechanisms include, limited gene expression of neutrophil collagenase and epithelial gelatinase, prevention of alpha1- antitrypsin degradation, and removal of reactive oxygen species (ROS) (24, 127).

**2-3-2) Surgical Interventions**

Eye injuries because of contact with sulfur mustard are divided in two categories; acute, chronic and delayed. Most victims with acute clinical manifestation recover to a completely normal state after a few weeks, but chronic and delayed mustard gas lesions usually cause developed and permanent decrease in visual acuity and even blindness. One of the chronic and delayed complications is mustard gas keratopathy (MGK) that includes about 0.5% to 1% in cases with severe exposure. It usually happens and is hard to treat this condition (28, 128). Other findings include: disabled corneal sensation, recurrent/resistant epithelial erosion, damaged limbal vasculature, neovascularization, corneal irregularity and thinning, descemetocele and sometimes perforation (28, 31, 128).

**●Tarsorrhaphy**

If thinning of the cornea is advanced in nasal or temporal zone with or without persistent epithelial defects (PEDs), medial or lateral tarsorrhaphy can be used to prevent the progress of corneal thinning (28, 31, 129). This procedure greatly reduces the symptoms of chronic irritation of the eye surface and the dry eye, and results in healing (31). This method is increasingly applied after stem cell or corneal transplantation(30, 129). 

● **Human Amniotic Membrane Transplantation**

Using amniotic membrane in conjunctival plastic surgery had previously been introduced(130). It is thinner and more tolerable to the patients. It is avascular, multilayered tissue with antiangiogenic, antiscarring and anti-inflammatory properties. Because it does not express antigens of histocompatibility, the membrane will never be rejected after transplantation. Using cryopreserved methods the useful effects of decreasing inflammation and neovascularization persist for a long time (131).

Amniotic membrane (AM) has many uses, which are either graft as an alternative in damaging ocular surface stromal matrix or as a patch (dressing) in preventing unwanted inflammation the result ocular surface damage. Of course, it can also be used in combination (131). Limbal stem cell deficiency can occur due to the degradation of limbal epithelial stem cells and/or limbal stroma (niche) inefficient (129). While a persistent epithelial defect (PEDs) is associated with partial limbal stem cell deficiency (LSCD), an amniotic membrane transplantation can be used. Even if it involves as large as 120° to approximately 360° in the limbus. The result in the eyes was a smooth and stable corneal epithelial surface without erosion or persistent epithelial defect, less stromal cloudiness and vascularization eventually. If LSCD is severe, the amniotic membrane transplantation is not helpful (23, 132). An important point is the use of suture in the corneal epithelial defect, which epithelium may grow under amniotic membrane. Therefore, instead of suturing using fibrin glue, this will prevent the formation of epithelium under amniotic membrane(133). Whenever severe eye irritation occurs with disturbing photophobia, which is followed by lipoid deposition in the cornea, superficial keratectomy associated with amniotic membrane transplantation becomes very useful (23).It should be noted that multiple amniotic membrane transplantation may be effective in the treatment of deep ulceration of the cornea and sclera (130).

●**Stem Cell Transplantation**

The patients with mustard gas keratitis (MGK) have problems such as irritation, redness, and tearing of the eyes with persistent epithelial defects (PEDs), dry eyes, stromal neovascularization, focal corneal thinning and ulceration, also loss of keratocytes and endothelial cells, and lipid and amyloid depositions. In this situation, they do not respond to conservative and regular treatments and may require stem cell transplantation (23, 30, 128, 129).

If total limbal stem cell deficiency (LSCD) involves only one eye, limbal conjunctival autograft transplantation can be achieved, but total LSCD may complicate both eyes, and limbal epithelial stem cells for allogeneic source can be used (134). Limbal stem cells are harvested from carriers, including parents, siblings, or children, known as living-related conjunctival-limbal allograft (lr-CLAL) or the cadaveric eyes known as keratolimbal allograft (KLAL) (135). Tissue harvested from one eye or two eyes from family members by lr-CLAL method is newer and genetically closer to cadaver by KLAL methods. But a KLAL graft is more available and it has more stem cells. In addition, it is weak stem cells and reject chronically (23). It is noteworthy that adjacent to the limbal areas are the thinnest parts of the peripheral cornea with epithelial defects. These areas can be selected as surgical sites (23, 129). Due to its partial, bilateral and non-symmetrical LSCD, and the difference in severity of the involved quadrants, 360- degree coverage of the limbal region with a graft that is inessential (129). To prevent rejection of corneal grafts, immunosuppressant drugs are used. For prophylaxis and treatment, these drugs including topical and systemic steroids (prednisone; 0.5 mg/kg/day P.O), cyclosporine A; 2.5 mg/kg/day P.O, tacrolimus (FK506); 0.2 mg/kg/day P.O, mycophenolate mofetil; 2gr/day P.O are used (136).

●**Corneal Transplantation**

If LSCD is not severe and central corneal opacification caused a decreased visual acuity, penetrating keratoplasty (PKP) or lamellar keratoplasty (LKP) has been used (28, 30). Many studies pointed to corneal layer damage in chronic and delayed mustard gas keratitis. These abnormalities were observed in all corneal layers, but severity of anterior to middle parts is more than the posterior parts(128). Deep lesions need a full-thickness graft (23). In the eyes with severe corneal thinning, large descemetoceles, and the possibility of corneal perforation is high, may require tectonic PKP, and in patients with small descemetoceles may carry out tectonic LKP (23, 30).

One of new procedures is called deep anterior lamellar keratoplasty (DALK) or deep lamellar keratoplasty (DLK). DALK can be presented to patients with normal endothelium without deep posterior stromal scars to descement membrane. It performs the deepest level to decrease scarring, also has same thickness of the posterior layer, a donor tissue of suitable thickness, good arrangement of the edges of the graft, and identical traction of the sutures (137).

The benefits of the DALK include: decreased endothelium graft rejection (138), maintaining of host endothelium with minimal surgical trauma and cell count (139, 140), effective visual rehabilitation to PKP (141), as well as less intraoperative and postoperative complications (139). DALK is better to PKP for protection of endothelial cell density. It is a long-term graft survival time, also it is safer than PKP because is an extraocular method (137). Corneal transplantation can be performed with limbal stem cell transplantation at the same time, but experts are recommended to perform that for at least 3 months after stem cell transplantation. Because of ocular surface damage and keratopathy caused by sulfur mustard, corneal transplantation is at high risk. Therefore, delaying the corneal transplantation will reduce the chance of rejection (23, 129).

◘ **2-4)** **Management of the bone marrow suppression**

Many experiences have been described in relation to the sulfur mustard damages, one of which is the suppression of bone marrow(104). Bone marrow damages comprise complete reduction of the granulocyte locations and degraded changes in megakaryocytes, leading to aplasia. Moreover, sulfur mustard deeply affect other cells such as lymphocytes, platelets, and red blood cells. Leukopenia and neutropenia may result from bone marrow suppression or leukocyte dispatch from the blood stream to infection (142).

Hematopoietic growth factors such as granulocyte colony stimulating (G-CSF) and pegylated form of G-CSF (Peg-G-CSF) have been approved by Food and Drug Administration (FDA), protecting the bone marrow from the toxic effects of sulfur mustard. Peg-G-CSF has a longer half-life and more retained duration of action than G-CSF. Peg-G- CSF improved the sulfur mustard-induced neutropenia as fast as or faster than G-CSF, but this effect was not completely on hold. These two compounds, in addition to reducing the duration of induction of neutropenia due to sulfur mustard, can reduce antibiotic use in these victims following secondary infection, increased survival times, and reduced duration of hospitalization for these patients (104, 142).

**◘2-5) Gene therapy**

The development of new treatment strategies, such as immunotherapy has been considered to increase the effectiveness of treatments by targeting malignant cells through various mechanisms(143)**. **Gene therapy has been the recent method to treat a numerous range of genetic disorders, specially cancers, that occur by the insertion of a manipulated gene into the defective cell. This includes two main strategies: (1) gene replacement, in fact the delivery of a normal gene to the cell, for modifying cell function through correcting the functional protein, and (2) gene silencing, a process in which an antisense RNA to silence an overexpressed gene. This action is performed to suppress the transcription in the cells by connecting to a special mRNA location through base pairing in DNA molecules. Delivering healthy and effective genes to target cells can be done by physical (ultrasound and electroporation), chemical (lipid and polymer-based gene vehicles), and biological methods (bacteria, viruses, and exosomes) (143-145). These strategies can specifically affect tumor cells without an important effect on normal cells. Hence, they bring better quality of life for the patients. However, gene therapy, cancer vaceiness and epigertic agents could not completely prevent relapse of complications in some cancers such as squamous cell carcinoma, melanoma, lung, prostate and hematopoietic malignancies. Table 2 shows factors affecting genetic profile, cancer vaccines, epigenetic drugs with dose and general side effects(143).

**Table 2:** The proposed drugs (genetics, cancer vaccines, epigenetics) in patients exposed to sulfur mustard, which have caused a type of cancer with their doses

* Genetic therapeutics		
**Medicin name**	**The dosage and method of its implementation**	**Author(s) or Sources-Year-Reference(s)**
-Gendiceine	It is a dose of 1.0ᵡ10^12^ viral particle intratumoral injection once a week for eight weeks, before or simultaneous radiotherapy 2 Gy per fraction, five fraction a week to a total dose of 70 Gy.	Zhang et al.-2005,(146) Ma et al.-2008,(147) Zhang et al.-2018,(148)
-Rigvir	-After surgery, was administered 2 ml intramuscularly for sequential days. Next, after 4 weeks, it was repeated for three consecutive days and continual about 1 month later. This regimen will be the first year. - Then, every 6 weeks during the first half of the second years every 2 months during the second half of the second year. - Thereafter, every 3 months during the third year.	Donina et al.-2015,(149) Babiker et al.-2017, (150)
-Oncorine	It is a dose of 5ᵡ 10^11^ -1.5 ᵡ10^12 ^viral particle, intratumoral injection per day for 5 days. Treatment repeated every 3 weeks. Note: All patients should receive at least 2 courses of chemotherapy.	Xia et al.-2004, (151)
-Talimogene laherparepvec, or T-vec (Imlygic, Amgen)	It is injected subcutaneously or intralesional so that first dose is 10^6^ plaque forming unit (PFU)/ml, then after 3 weeks, 10^8^ plaque forming unit (PFU)/ml every 2 weeks for at least six months.	Andtbacka et al.-2014, (152) FDA-2015, (153)
* Cancer vaccine		
-Cimavax-Epidermal Growth Factor (EGF)	After 4 to 6 weeks from the end of the first chemotherapy session, it is injected 200µg over 4 sites intramuscularly (50 micrograms per each deltoid muscle and each gluteus muscle) every 2 weeks for 4 doses and then every month	Cheng & Kananathan-2012, (154) Rodriguez et al.-2016, (155)
-Sipuleucel-T (Provenge^TM^)	It is IV infused a minimum of 50 million autologous CD54+ cells activated with Prostatic acid phosphatase (PAP) or Granulocyte-macrophage colony-stimulating factor (GM-CSF) in 250 ml of lactated Ringer solution , every 2 weeks, for three doses	Kantoff et al.-2010, (156) Anassi & Ndefo-2011, (157)
* Epigenetic therapeutics		
-Azacitidine (Vidaza^TM^)	It is injected subcutaneously or intravenously 75 mg /m^2 ^ for 7 days, next every month ( Based on its effectiveness, it will be adjusted during subsequent doses).	Kaminskas et al.- 2005 a, b, (158, 159)
-Decitabine (Dacogen^TM^)	Two regimes are recommended for it: Regimen1: It is IV infused 15 mg/m^2^ tid for 3 days, with intervals of 6 weeks Regimen2: It is IV infused 20 mg/m^2^ daily for 5 days, with interval of 6 weeks	Rahmani & Abdollahi- 2017, (143)
-Vorinostat (Zolinza^TM^)	-400 mg is given orally once a day, preferably with food. -If necessary to reduce toxicity: 300 mg once daily or 300 mg once daily for 5 consecutive days per week. Treatment will be continued until disease progression, unacceptable toxicity, inefficiency, or dissatisfaction with the patient.	Mann et al.-2007, (160)
Romidepsin (Istodax^TM^)	-IV injected 14 mg/m^2^ over a 4-hour on days 1, 8 and 15 of a 28-day cycle. - It is repeated every 28 days if it is helpful and tolerable to the patient. - To control undesirable drug reactions, the medication will either be discontinued or interrupted with or without reduction to 10 mg / m^2^.	FDA-2014, (153)
-Belinostat (Beleodaq^TM^)	-It is IV infusion of 1000 mg/m^2^ over 30 minutes once daily on Days 1-5 of a 21-day cycle. * Each vial also contains 1000 mg L-Arginine, USP as an inactive ingredient. **With 9 ml of sterile water for preparation, then diluted with 250 ml of sodium chloride 0.9% for intravenous infusion. -To control undesirable drug reactions, the medication will either be discontinued or interrupted with or without dosage reduction by 25%.	FDA-2014,(161) Lee et al.-2015, (162)
-Chidamide (Epidaza^TM^)	-It is administered orally 30 or 50 mg twice a week for 2 weeks, followed by 1 week of rest. - The total drug is taken at two orders, 120 mg and 200 mg for a 3-week period, respectively.	Shi et al.-2015, (163)
-Panobinostat (Farydak^TM^)	-It is administered orally eight three-week cycles. Each cycle includes; 20 mg, taken orally once every other day for 3 doses per week (on Days 1, 3, 5, 8, 10, and 12) of weeks 1 and 2 of each 21-day cycle. *With regard to the clinical benefits, 8 cycles continue, unless the severity of the disease is resolved or significant toxicity occurs. **Some sources point out that with this drug, prescribe bortezomib 1. 3 mg / m^2^ intravenously and dexamethasone 20 mg orally.	San-Miguel et al.-2014, (164) FDA-2015,(165) Moore-2016, (166)

## Conclusion

After the synthesis of mustard compounds (e.g. sulfur mustard) in the nineteenth century, it has been frequently used as incapacitating or death agent in wars in the last century, and may be used in war conflicts and terrorist attacks in the near future or not far away. Sulfur mustard is a toxic agent with severe effects on various systems of human body. Sulfur mustard with a number of pathogenic mechanisms referred to with certainty, such as DNA alkylation, NAD depletion, inactivation of glutathione, and leading to loss of preservation against oxidant stress, e.g., GSH depletion, increase in cellular ROS level, causing damage to various organs of the body. Different systems in the human body are affected by this chemical warfare, but skin, respiratory, and eyes are the main systems undergoing the provocative effects of sulfur mustard. When rapidly absorbed by human body, it quickly damages the cell proliferation in the bone marrow, and severely suppress the immune system. Their effects will appear several minutes to weeks even years. The patients and their countries involved will have many economic, social, psychological problems, and medical costs for long period of time. Therefore, authors of this paper wish that international organizations such as the organization for prohibition of chemical weapons (OPCW), the United Nations (UN), the World Health Organization (WHO), the Red Cross and the red crescent, and any international organizations related this point, also Non-Government Organization (NGO), not only approved the prohibition of the use of chemical weapons, but also in practice and deal with any country that uses these chemical weapons. It is hoped that in the near future, we will see the demolition of all destructive weapons globally, and that the slogan of peace, friendship and kindness to each other, bring out the best in us, because there may not be tomorrow for anyone of us. 
